# *In Loco Parentis*, Corporal Punishment and the Moral Economy of Discipline in English Schools, 1945–1986

**DOI:** 10.1080/14780038.2018.1518562

**Published:** 2018-09-11

**Authors:** Andrew Burchell

**Affiliations:** Department of History, University of Warwick, Coventry, UK

**Keywords:** Education, corporal punishment, teachers, parents, children

## Abstract

This article uses debates surrounding teachers’ *in loco parentis* position to explore the social and cultural responses to school corporal punishment in post-1945 English schools. Analysing materials produced by educators and campaigners, it argues that retentionists conceived of their right to inflict physical chastisement as one based on an imagined and discursive status as a parent. This was challenged by opponents who stressed not only the severity of the practice but sought to directly counter the view that parental rights should be automatically delegated to teachers. Whilst the abolition of corporal punishment was ultimately a consequence of an ECHR ruling, it is suggested that it can also be read as the culmination of a longer shift in the status and forms of parental rights in twentieth-century Britain.

When the 1938 Home Office Departmental Committee on Corporal Punishment administered in judicial settings (known after its chair as the Cadogan Committee) recommended the abolition of birching for juveniles, its argument was framed through an opposition of the penal regime to the domestic and school interior. The report’s authors believed that there subsisted a difference in kind, not merely in severity, between the relationship of a misbehaving child to a parent or teacher and that of a young delinquent to a police officer. Placing the school alongside the home as an environment where a ‘continuing’ interaction between punisher and child existed, it was suggested that physical chastisement’s harmful psychological effects – so pronounced in the artificial penal setting – were counterbalanced by a teacher–pupil relationship characterised by ‘respect and often affection’.^1^ Consequently, the committee advocated the abolition of judicial birching while framing its decision in such a way that ‘corporal punishment as a means of enforcing discipline in the home or in school’ remained beyond its scope.^2^ Such wording implies a greater consensus in favour of school corporal punishment than was actually the case. Throughout the twentieth century – until its definitive abolition in maintained (state) schools in 1986 – the practice of caning was the subject of increasing polemics, which sought to challenge and disrupt the imagined discursive relations created in sources such as Cadogan between the teacher and the parent. By positing that the ‘parental’ understanding of discipline explicitly articulated in this Home Office report mirrors that of teachers’ own professional attitudes towards corporal punishment in post-war Britain, I contend that the discourse of teacher-as-parent was used by educators and their representatives to ascribe positive meanings to the practice of disciplinary force. The question of how far a teacher should enjoy disciplinary powers delegated *in loco parentis* therefore became a key battleground in discussions of physical punishment after 1945, amid mounting calls for its removal as a legitimate sanction in state primary and secondary schools.

Tracing the disciplinary uses of *in loco parentis* by teachers and others interested in educational theory and practice over the mid-twentieth century can also shed light on the limitations of both the concept’s discursive construction in addition to what can be considered the moral economy of discipline subsisting between the trinity of teacher, child and parents. Moreover, the later part of the period, characterised by the emergence of targeted campaigning organisations, provided new means for – often middle-class – parents to express discontent and highlighted the emergence of education as a more consumer-focused concern, as Peter Mandler has recently argued.^3^ Groups like these were successful, as Moira Maguire and Séamus Ó Cinnéide have persuasively suggested in relation to corporal punishment in Irish schools, in both emphasising the brutal, sadistic nature of corporal punishment whilst progressively situating parents as the natural source of discipline over the educator.^4^ The near-absence of a similar discussion of corporal punishment and its polemics from the historiographies of British education, teacher professionalisation and the gendered relations of the classroom is all the more striking when we consider the centrality of corporal punishment – as a threat and a practice – to everyday experiences of education throughout twentieth-century Britain. Focusing on interactions between state agencies (the school board and local authority) and the working-class families who were the subjects of compulsory education in the late-nineteenth, school corporal punishment has been integrated into a narrative of working-class youth’s experiences of oppression and resistance.^5^ Meanwhile, historical research into the extent of the practice’s use, and the meanings ascribed to it, before the era of compulsory schooling is even more sparse.^6^

This dichotomous and abrasive perception of schooling and class relations has recently been revised, particularly in relation to the inter-war period when schooling became more embedded in the national consciousness across class lines. Hester Barron, Siân Pooley and others have reconceptualised such themes more in terms of fluid ‘interactions’, especially as far as intermediary professions such as teachers and social workers were concerned.^7^ Likewise, Barron and Christine Wall have drawn attention to configurations of schools through discourses of *in loco parentis*, which enabled teachers to present themselves as counterbalances against poverty and deprivation.^8^ The inter-war period is also identified as a turning point by Deborah Thom in her work on the use of corporal punishment in judicial and home settings.^9^ Judicial birching declined considerably after 1918 and Thom asserts that through debates played out among psychologists and in the pages of the 1967 Plowden Report, the school ‘gradually became a place in which corporal punishment was first tightly monitored and then abolished’.^10^ In a more recent foray into these themes, she implies that the vast majority of local education authorities (LEAs) had begun to dispense with the practice by the ‘permissive’ 1960s.^11^ Although Thom is correct to point to the role of campaigners, government reports and psychological ideas in delegitimising the practice, this takes little account of parallel shifts in constructions of the teacher’s authority. Moreover, contemporary polemics over the reliability of official records and brutality appear to provide ample evidence for a much longer continuation in frequency and severity than previous research has acknowledged.^12^ In other words, while there may have been a shift away from its use against younger children and towards adolescents – with one teaching union admitting in 1983 not only that the cane was still prevalent but that it was ‘in the secondary sector that corporal punishment has continued most in use’ – it is the categories of class and gender, as much as age, which are implicated in polemics over corporal punishment.^13^

While the image of the ‘beater’, pathological or otherwise, was virtually always presented as a masculine one, there is evidence from oral histories that inter-war female teachers (while occasionally being shocked by the excesses of male colleagues) did nonetheless support and have largely unproblematised recourse to corporal punishment.^14^ As teachers came to articulate their professional powers and rights in terms of relations between school and home, I will argue that this drew upon the legal notion of *in loco parentis* to justify their parental self-image. This nonetheless had a longer history, and the idea of the female teacher as a ‘mother made conscious’ is one that, as Carolyn Steedman argued, dates from at least the nineteenth-century Romantic movement.^15^ Furthermore, as we shall see, male teachers could develop their own versions of maternal theories, with Laura Tisdall and Laura Carter both finding evidence for male teachers claiming a more paternal role for themselves in dealing with ‘problem’ male pupils.^16^ It is only comparatively recently that attention has begun to alight on the construction of personal, subjective identities in relation to the classroom and the children taught, with earlier professional histories having elided these aspects and focusing instead on organisational structures and key points of dispute with authorities.^17^ While not wishing to underplay the significance of the heterogeneity and diversity of unions groupings, their relative homogeneity on the issue of corporal punishment and *in loco parentis* should gave pause for thought as to whether both served as markers of professional identity.^18^

It is for these reasons that trying to map changes in frequency over time is problematic. As Laura King identifies in relation to domestic masculinity and fatherhood in twentieth century Britain, there are two broad stages of decline in home-based corporal punishment observable in oral histories; a first move in the 1950s and a second in the 1970s both complemented by a further shift away from beating and towards smacking.^19^ Subtle shifts such as these emphasise the role of subjectivity in forming views as to the suitability and use of punishment. Moreover, while this can be understood by interviewees as a reaction against an older, more disciplinarian form of domesticised masculinity there remains a limit on assessing the true extent of these practices through analysis of professional and public discourses alone.^20^ As King notes, there was a ‘tension between diversity on public attitudes … and the editorial line’ of most newspapers or parenting guides.^21^ It is these divisions which may account for the fact that while parental authority was appealed to in sources on both sides of the school punishment debates, as well as all the nuances in between, parental punishment was always an unspoken and strategic silence.

Finally, it should be noted that I employ ‘moral economy’ here as shorthand for the interpersonal relations governing the teacher and child in the classroom setting, particularly as it relates to perceptions of the reciprocal rights and responsibilities of each.^22^ Contrary to some philosophical and economic analyses, which assert that applying the term beyond monetary relations is to denature its meaning, I would argue that the term as originally delineated by E P Thompson implies a more cultural relationship defined by reciprocity.^23^ This rendered it open to challenge by the eventual refusal of either party to honour the traditional terms of the agreement in question. This seems especially pertinent to punishment because *in loco parentis* was a principle in common, rather than statute, law and teachers were therefore forced to navigate tradition and precedence to justify the continuance of their right to use disciplinary force. In employing the term to designate alterations in relationships as a consequence of social and cultural changes, this essay aligns its understanding of the moral economy of *in loco parentis* more towards that of the historian of science Lorraine Daston in her analysis of the legitimising value attached to scientific objectivity through an affective lens.^24^ Expectations of what the reciprocal roles in the classroom setting should be therefore inflected understandings of how to punish.

Following an historical overview of *in loco parentis*, I will offer a summary of the processes culminating in the abolition of corporal punishment in 1986. A second section will then examine how teachers’ disciplinary authority was constructed in psychological and pedagogical texts through an appeal to the metaphors and legal institutions of parenthood. This will make use of advice manuals produced by teachers and psychologists, as well as internal union documentation, correspondence and submissions to government committees and departments. The final section considers the challenges to this moral economy of classroom order and discusses the contested forms of parental authority in relation to the child revealed by them. Because *in loco parentis* is a legal concept, this article is concerned solely with its understandings in relation to the legal jurisdiction of England and Wales. For clarity of argument, it also avoids consideration of how *in loco parentis* discourses operated where parents were either absent (children in care homes or boarding schools) or the child was separated from them for disciplinary reasons (such as approved schools).^25^

## *In loco parentis* and corporal punishment in English law

*In loco parentis* permitted teachers in England and Wales to employ a common-law defence of reasonable chastisement against any charge of using disciplinary force, which included slapping or cuffing with the hands as well as caning, administered to their pupils for the purpose of discipline or correction. The most literal definition of *in loco parentis* characterises it as ‘in the place of a parent’, and the principle was central to the relationship between all of the parties involved in the raising of children from the apotheosis of the elite boarding schools in the mid-nineteenth century onwards. As one textbook guide to case law for teachers phrased it, *in loco parentis* resulted from the legal assumption that a parent, ‘when he places his child with a schoolmaster, delegates to him all his own authority’, including that of the right to administer physical correction.^26^ Such delegation was limited to what was ‘necessary for the welfare of the child’ and to the ‘duty … of a careful father’.^27^ Assessing the fairness of correction given according to this principle also situated the parent as its standard. It had to be ‘moderate, not dictated by bad motive, such as is usual in the school, and such as the parent of the child might expect it to receive if it did wrong’.^28^ One outcome of this was that the extent of corporal punishment reasonableness was largely determined negatively, with cases resulting from punishment ‘gone wrong’ defining the boundaries of the permissible.^29^ *In loco parentis* as a legal doctrine therefore governed teachers’ duties as they related to the law of negligence and welfare; responsibilities to which the exercise of corporal punishment might be annexed as a necessary expedient. It was based on parental rights but existed independently of them, and parents could not refuse to delegate their authority. It was also, as the above quotations demonstrate, a notion which applied to all teachers regardless of gender but, in common with other sources, contained an implicit bias towards male pronouns and male examples (the ‘father’ as a natural source of discipline being a prime example).

Largely neglected as a concept by historians working with childhood and education, analysis of *in loco parentis* has been most commonly addressed in works on universities rather than schools.^30^ Originating in the early-modern period, the principle that adults other than a parent enjoyed some responsibility for the welfare of a child would take on added significance during the nineteenth century as primary education became compulsory after 1880. Applying such a legal relationship in local authority day schools, where children’s time was divided between home and the institution rather than the totalised site of the elite boarding school, lies at the root of historiographical debates over the extent of working-class ‘resistance’ to educational expansion.^31^ Yet, as Rob Boddice has shown, the concept was even contested in the public schools, although usually over efforts to exclude their male pupils from pursuits their parents regarded as healthy and manly.^32^ It might therefore be concluded that the doctrine has always been debated and refined as much outside of the law courts as within them.

Some of the initial resistance to compulsory education declined in severity during the early part of the twentieth century. This was partly the result of generational shifts and partly of social changes in attitudes. Early-twentieth-century parents had, for instance, benefited directly from education themselves. Moreover, the education system had become more embedded in the emerging notion of state welfare. Nevertheless, as Barron shows, there was still scope for disagreement between teachers and parents.^33^ One crucial difference with the earlier period, perhaps, was that with education now established as a mass phenomenon, the first national and coordinated campaigns against the practice of disciplinary violence could also emerge. Initially, opposition gained ground among fringes of the teaching unions and radical educationists during the inter-war period. Figures such as A S Neill or Ethel Mannin (a key populariser of Neill’s ideals) were particularly vocal in viewing caning as a danger.^34^ A short-lived Committee for the Abolition of Corporal Punishment in Schools was established just after the war, although it appears to have become inactive by the early 1950s and its archival record has remained largely untraceable (at least for this researcher).^35^ The intervening period saw the issue fall from public and legislative attention. Following the 1967 Plowden Report into primary schooling, and the recommendation contained therein to abolish its use for pre-secondary children, the issue emerged once again as a subject of polemic.^36^ A new campaigning organisation, the Society of Teachers Opposed to Physical Punishment (STOPP), was created in 1968 by the teacher Gene Adams, with the support of the progressive education group CASE (Campaign for the Advancement of State Education), to promote the implementation of the Plowden recommendations and their extension to secondary schools.

STOPP enjoyed early success. Although *in loco parentis* served as a legal justification for corporal punishment, the power to inflict it could still be removed or limited by individual LEAs who controlled both the regulations of individual schools and their teachers’ terms of employment. A minority of local authorities were persuaded to abolish the practice unilaterally, with the Inner London Education Authority gradually ending its use for all age groups by the late 1970s. Nevertheless, the majority saw no reason to revise their punishment regulations until the early 1980s.^37^ Instead, it would not be until after 1982, when the European Court of Human Rights in Strasbourg offered its verdict on the validity of parental objections to school chastisement, that government policy and teaching union attitudes abruptly seemed to shift against this status quo and towards one of enforced prohibitions.^38^ *Campbell and Cosans v United Kingdom*, brought by two Scottish mothers, centred around parental oppositions to the practice and their effects on *in loco parentis*. It is worthy of note that the parents’ first ground of appeal against corporal punishment (that it constituted a ‘cruel and unusual’ punishment) was actually rejected by the court, although the judges sided with their secondary argument that opposition to corporal punishment should be respected by schools as a parental ‘philosophical and moral’ conviction. The archives of teaching unions, STOPP as well as the National Archives reveal that integrating the ruling effectively into UK legislation provoked several years’ worth of negotiations between the government and teachers. Initially, the plan was to allow individual parents to ‘opt-out’ of having their children caned, although the mechanics of this proved difficult to devise.^39^ Teachers deplored the idea of inequalities between individual pupils, while debates about whether the system should be ‘opt-out’ or ‘opt-in’ took up the efforts of civil servants.^40^ As a consequence, previously retentionist institutions such as the National Union of Teachers (NUT), Assistant Masters and Mistresses Association (AMMA) and National Association of Schoolmasters (NAS-UWT) passed conference resolutions supporting total abolition over partial opt-outs.^41^ Teachers’ right to use physical chastisement, although not their *in loco parentis* status, was repealed by statute in 1986.^42^

The verdict is therefore significant because it negated both the child’s right not to be hit and the teacher’s right to employ force against any child. Parents’ own disciplinary authority, meanwhile, was validated as the primary source of punishment. *In loco parentis* was reconfigured as a delegation of authority, and the debate refocused on how far such delegation could be legitimately withheld. The notion that parental views on the appropriateness of corporal punishment should override that of the teacher legally ‘acting as a parent’ was not, however, a new idea in the context of the ECHR judgement. This was reflective, perhaps, of an increasing emphasis on parents’ rights in post-war constructions of the child’s right to an education.^43^ In any case, it was a substantial reimagining of *in loco parentis* from that of teachers and one that is worthy of further historiographical development.

## Acting as a parent: the teacher–pupil relationship, gender and class

Over the course of the twentieth-century, experts in psychology, as well as teachers and educational commentators, became increasingly interested in the emotional, lived realities and childhood and consequently in the relationships children forged with the adults responsible for them.^44^ The stress placed on the dynamic nature of child–adult relationships found its most notorious post-war iteration in ‘Bowlbyism’ and parental attachment theories, with a particular and noteworthy focus on the mother, although fathers could equally play a role, as concerns over the effects of absence on behaviour and delinquency testify.^45^ Yet, classroom pedagogy and particularly the relations forged between teacher and taught were equally important for educational psychologists of a variety of intellectual persuasions. Writing in the inter-war period, one psychoanalytically minded educationist found *in loco parentis* ‘absurd’ and a ‘pernicious’ example of ‘jargon’ in educational theory.^46^ For most twentieth-century commentators, though, *in loco parentis* was more than a doctrine in law; it was an idealised statement of the circumstances which ought to subsist between the two halves of the classroom dynamic. The secondary-modern teacher, Richard Farley, in his advice manual to colleagues who might be faced with the ‘“difficult” adolescent in socially depressed industrial areas’ (1960) used the concept to present a path for the (male) classroom teacher that merged pragmatism with romanticism.^47^ Teachers should endeavour to become ‘*in loco parentis* in the true meaning and spirit of the phrase’, casting the educator somewhere between what he termed a ‘confidant’ and a substitute parent who, amongst other things, might take troublesome adolescents on impromptu visits to sites of interest at weekends.^48^ Rex Bowley, his contemporary and the author of *Teaching Without Tears* (1961), adopted a similar, if more restrained, approach: urging teachers to understand the legal framework within which discipline operated, seek to establish their authority among pupils and then proceed to seek their confidences. The culmination of this, in an interesting gendering of terms, was that the teacher ‘can afford to allow [pupils] to tease him, as a father will allow his children’.^49^ By the late 1950s, as King argues, the trope of the heroic, ‘substitute’ father (often a school teacher or policeman) had become a fixed one in literature and films depicting juvenile delinquency.^50^

These are, of course, male examples and published writings by female teachers on these themes are very rare. What is interesting are the ways in which, as men, they tread a line over corporal punishment. For Bowley, it was best avoided, whereas Farley remained more ambivalent, feeling that it might work in some cases but equally cause irreparable harm in others. Central to both was the importance of relationships, and the need earn respect from pupils by exuding paternalistic authority. In this regard, it is revealing that some of the harshest criticism in Farley’s work is directed at single and ‘working’ mothers who, he claims, ‘spoil’ their sons with gifts and pocket money and lack the intuitive male understanding of how to motivate a boy through willpower and respect.^51^ They posit a different form of masculinity than that to which the boy is exposed in the home. These diverse voices found an unlikely ally in a shifting psychological and cultural understandings of freedom in post-war child psychology, a movement towards a belief in ‘freedom through discipline’ which saw the establishment of order as a vital prerequisite for the expression of personal liberty rather than a hindrance to it.^52^

Farley suggests that *in loco parentis* entailed a belief that educators should wield the cane or strap, not with impunity, but with the same alleged tenderness and utilitarian concern for individual and group welfare as a parent. This was an appeal to a moral economy of gendered order, in which correction readjusted a perceived fault in the relationship between teacher and taught. Yet there were a myriad of different positions emerging on corporal punishment at this time, all of which seemed to value appeals to different interpretations of the classroom moral economy. Collectively, these developments ensured that all sides in corporal punishment debates were fixing their attention not only on the actual sanction but equally on the situations and contexts in which it was imposed. The Cadogan Report, as we saw at the beginning of this essay, argued that the harm of judicial flogging derived from its essentially ‘impersonal and cold-blooded’ nature, which contrasted to the ‘affection’ of the schoolmaster.^53^ The emotional bond maintained order, ensured that punishment could not be transgressive or cruel and, for teachers, resisted any threat to corporal punishment by allowing them to claim the same rights existing in the natural family environment.

The persistence of this kind of attitude throughout the period under scrutiny is perhaps best illustrated by noting the similarities in positions on punishment across union divides. Then, as now, the numerically largest teaching union in England and Wales, the NUT was characterised by a mixed membership, in terms of gender, primary and secondary staff, as well as teachers at different professional grades. Its emergence at the end of the nineteenth century as the representative institution of teachers in local authority schools meant that its staff were the most likely to come into contact with working-class children, unlike the organisations oriented towards independent and grammar school staff, such as the Assistant Masters Association (AMA) and the Association of Assistant Mistresses (AAM). The NUT submission to the 1960 Barry Commission, convened in the early 1960s to re-examine the question of corporal punishment as a criminal sentence, maintained a conception of school punishment as occurring within a positive affective framework.^54^ Echoing Cadogan, the NUT argued that the potential harms of judicial corporal punishment derived precisely from its essentially ‘impersonal’ nature, contrasted to the teacher’s privileged position:

Corporal punishment in schools is not an isolated matter divorced from what goes before and what comes afterwards. It is given in complete knowledge of the pupil, his background, his temperament, and his general social behaviour and attitude … the teaching profession would doubt the value [of corporal punishment] except when it is administered by a wise parent or a teacher acting *in loco parentis*.^55^

Juxtaposing the image of the ‘wise parent’ with that of the ‘teacher’ (whose position is stressed as a legal one through the direct invocation of *in loco parentis*) is here rendered central to claims for professional knowledge of the child’s needs in a unique, holistic way. The evidence equally sought to imply that the teacher’s strength as a force for discipline derived from a relational position, reassuring the reader that children were only punished in a way appropriate to them as individuals. More revealing, perhaps, is the fact that much of this NUT document had been drafted in 1948 and remained the standard model (often with a few minor contextual adjustments) for several punishment-related official and unofficial submissions until at least the mid-1960s. This included the response to the Plowden Committee on the primary school in 1965, and a variety of individual enquiries from journalists or researchers regarding the union’s position on the issue.^56^

The continuity of the NUT’s position is not solely reflective of organisational inertia or inherent conservatism, then, but rather a refusal to see the practice as problematic within the context of its professional and parental status. Responding to Plowden’s recommendation that corporal punishment be abolished for primary-school-age children, the NUT could thus observe that teachers had an awareness of children’s environmental and emotional limitations, coupled with a concern for both the individual child and the ‘needs of the whole class’.^57^ The skill required to resolve this dual, almost paradoxical, role was implied to be at the heart of the teacher’s status as a well-trained yet caring professional. A letter from the AAM to a representative in Wales over a decade later repeated similar arguments, noting that it had been in decline for several years and that it was the organisation’s hope that it ‘should grow out of schools gradually’.^58^ There is certainly something in these arguments of professional convenience, and teaching unions were undoubtedly implicated in maintaining the status quo. Yet, the concordance in professional views seemingly across demarcating categories like gender and, arguably, class reveals more of a unified perspective based on constructions of classroom relationships.

Teachers were aided in this line of argument by the fact that a variety of psychiatric and mental health organisations, in their own depositions to the Plowden Committee, had supported a framing of *in loco parentis* in precisely these psycho-dynamic, affective terms. Simultaneously, they were equivocal over how far causal links could be drawn between disciplinary beating and childhood maladjustment or neurosis. The National Association of Mental Health, for instance, noted that ‘the relationship between teacher and children […] is the most important factor’ in the maintenance of discipline.^59^ The British Psychological Society, meanwhile, also adopted a version of this cautious reasoning, considering that punishment had to be analysed in the context of ‘the total situation which led to it’ and the ‘personal relationships of the punisher and punished’.^60^ Such comments should not be read as evidence that the discourses surrounding corporal punishment remained unproblematised, either on psychiatric or social grounds. Limitations did exist but were often based on notions of propriety. Like many male commentators on the issue, who were generally supportive of the teacher’s right to have recourse to the practice, Farley instituted a strictly gendered division, rejecting outright the use of the cane against girls.^61^ This echoed received wisdom from psychologists, and the potential, and acknowledged, sexual subtext to flogging when carried out in a ‘heterosexual’ pairing of male teacher and female pupil (or vice versa).^62^ Its primary value was thus symbolic, a way of redressing the moral economy between teacher and taught when the reciprocal trust on which it should be founded had been ruptured by the actions of an unruly pupil. In such cases, the teacher was transfigured into an ideal parent, one who could employ soft parental power in a constructive dialogue between the educator and a child of the same biological sex.

Conversely, a focus on the circumstances of corporal punishment’s application was equally employed to argue against the practice, and the fact that teachers had imbibed this relational approach, given its centrality to oppositional arguments, offers an early insight into the tensions which surrounded the relationship approach to classroom discipline. As Peter Newell, a prominent member of STOPP and the editor of the self-consciously abolitionist edited volume, *A Last Resort?*, boldly declared, ‘physical punishment deeply affects teacher and pupil attitudes and the type of relationships possible within schools’.^63^ Arguments in favour of corporal punishment were forced to counter this by appealing to its usefulness in individual cases whilst taking account of causal, contextual factors and psychological theories about the familial disciplinary relationship.

The common bases for these ideas about discipline account, at least in part, for the intriguing similarities between views expressed by STOPP and the NUT on the subject of *in loco parentis* and its relationship to discipline. Part of STOPP’s campaign called for the replacement of caning with an increased reliance on the encouragement of greater ‘self-control’ or ‘self-discipline’ in children, which it alleged could only be achieved through influencing pupils’ lived environments, rather than externally through imposition by force.^64^ The best behaved children were alleged to work within a moral economy of parental affection whose distortion, or threat of diminishment, provided adequate sanction without the need for recourse to physical chastisement.^65^ The NUT evidence to Plowden likewise posited that ‘discipline’ required a new definition beyond its association with a fundamentally negative response to behaviour.^66^ As opposed to ‘a rigid system of conformity with an arbitrary set of standards of behaviour’, it was instead a question of preserving classroom relationships in which ‘the anti-social behaviour of the individual is not allowed to prevent the development of the other individuals in the group’.^67^ More explicitly than STOPP, however, the NUT submission connected these approaches to a vision of substitute parenthood, reaffirming from its first page that ‘[a] teacher is *in loco parentis* to the pupil’.^68^ Framed as a sometimes regrettable necessity in a caring pedagogical relationship, the use of corporal punishment afforded by this position was cast as a loving and child-centred act, and the language used survived corporal punishment. Following its conference resolution to oppose corporal punishment in 1982, after the Campbell and Cosans ruling, the NUT became all the more keen to stress the importance of reprimanding children in a positive way, one which involved promoting the basis of the school as a ‘basic caring relationship’, in the words of a union pamphlet.^69^

## Parental rights: contesting the moral economy of corporal punishment

Interpretations of *in loco parentis* thus furnished tensions between the various groups and campaigning organisations, even though (on closer inspection) almost all appeared to concur that some delegation of the parental role was not only necessary but integral – both for discipline and the mental well-being of all concerned. The rhetoric of a psychological and social dynamic between teachers and pupils nurtured an understanding of their legal relationship, which was crucial to the former’s perception of its right to discipline the latter. It is, however, worth reflecting on the bases of this parental authority, and its attendant contestations. The moral economy of the classroom, threatened by legal interpretation in the form of Campbell and Cosans, was equally challenged by alternative views of the teacher’s privileged position. Understandings of and appeals to *in loco parentis* structured a model of thought in which physical punishment was in some sense natural. But the precise expressions of this belief were influenced by the gender of the articulator, questions of class, and the political and ideological differences between individual unions.

Among the submissions on discipline received by the Plowden committee, for instance, that of the NAS was the only one not to invoke the psychology of punishment even once. Indeed, at one side of A4 paper, it was also one of the shortest submissions on the topic, outlining briefly the results of a survey it had carried out among its members. The fact that its submission was so short and that it privileged professional group opinion, with little need for appeal to external sources or alternative forms of knowledge for validation, certainly makes it unusual in the context of other union documents explored here. It was complemented by a more overt attack on educational theory, interpreting the marginally higher incidence of opposition to the practice among younger entrants to the profession to the fact that ‘attitudes change with increasing practical experience and with the realisation that idealistic theories are sometimes based on an insufficient knowledge of children’.^70^ The NAS had formally broken away from the NUT in 1922, in response to the perceived dominance of the latter organisation by female interests. An explicitly anti-feminist organisation, it was established to oppose equal pay and defend what it claimed as a special role for the male teacher against dangerous, feminising influences.^71^ Although these initially controversial issues had declined in importance for the NAS by the 1960s, with the lifting of the marriage bar and reviewed pay awards (culminating in its merger with the National Union of Women Teachers to form the NAS-UWT in the late 1970s), it still remained distrustful of educational theorising, as its Plowden submission shows. Concerned with classroom relationships as ‘real’ and concrete relationships – as Farley saw them – they warned the Secretary of State for Education, Shirley Williams, in 1977, about head teachers being ‘constantly catechised and criticised by fanatical abolitionists … for defending the reality of “in loco parentis”’.^72^

The AMMA, formed around a similar tome to the NAS-UWT by a merger of the AMA and AAM, adopted a different approach. In the aftermath of the Campbell and Cosans ruling, a union discussion document urged that the ‘teacher pupil relationship must give to the teacher the rights as well as the obligations of a good parent’.^73^ For AMMA, ‘neither unrestricted belief in corporal punishment … nor a call for its abolition, fairly represents the view of a large number of practising teachers’ and reflected its status as a ‘matter of professional judgement’ taken in a rounded knowledge of the child.^74^ For this reason, it advocated that any abolition ought to coincide with ‘changes in the law relating to young people and their parents’ responsibility for them’, noting that ‘[i]f teachers are to be *in loco parentis* only in a restricted sense, who or what shall stand in the place they have vacated?’^75^ The central concern articulated here was essentially one of balancing professional authority and knowledge in a continuum of a literal and spatial kind. If teachers’ ability to invoke *in loco parentis* were ‘restricted’, the authors suggest, a rupture would exist between the physical spaces of home and school. The NUT had been similarly convinced of an evolution in social attitudes more or less of a similar magnitude to this at an even earlier juncture (the late-1960s). But it merely cautioned that it would be hasty to employ such ideas as the basis for policy which would only serve to undermine the ‘wise tradition’ – what might be otherwise termed the classroom moral economy – in which ‘the running of the school’ was a teacher’s responsibility and defended as such through constant repetition of the tropes of ‘professionalism’ and trust. ‘Interferences’ from the state, they asserted, ‘will only hinder these professional developments. […] The record of professional thinking over the years shows that this is an issue which can be safely left to the teachers’.^76^

These attitudes complement the findings of oral history research, which have identified the attention paid by teachers in their own life-narratives to what Philip Gardner has termed their ‘working relationship’ with pupils.^77^ Farley expressed this understanding of the dynamic, moral economy of the classroom when he wrote that ‘my experience has taught me that although the cane can work (not always), the best approach to an anti-social thug is the personal approach, the gradual building up of a reputation for being “a decent bloke, but a right b------ if you go too far”’.^78^ The situationality of this made him distrustful of making prescriptive judgements other than those supported by pragmatism, to the point of interpolating the reader’s confusion directly:

The novice may be rather mixed up by now. Sometimes the cane appears to be advocated, sometimes not. One speaks of affection one minute and caning a boy hard the next […] I am trying to be severely practical and am only discussing attitudes and remedies which work in our present legal and social framework. It is utterly useless for me to advocate what ought to be, or even what ought not to be, because a teacher has to take his classroom and environment as he find it.^79^

Farley’s response is therefore governed by a similar desire to that in the AMMA policy document. He wishes to accept the ‘limitations’ of power, not simply over external in behaviour (the events of differing practices of childrearing or the prevalence of delinquency) but arguably also in terms of space. The school site delineated the physical boundaries of a teachers’ *in loco parentis* authority, as distinct from the seemingly unbounded exercise of parental power.

Challenging the moral economy thus required opponents of the practice to expose these contradictions within its sense of space. By the late 1970s, these ideas had morphed into two major threads and tropes of STOPP’s campaign. The first was what they perceived as brutality; the second was promoting parental opposition to the automatic ‘delegation’ of their power under *in loco parentis*. Not infrequently, however, the two were connected. For example, one teacher contributor to *A Last Resort?* explored the ‘ritualised’ nature of corporal punishment, which he felt rendered it so violent and psychologically damaging as to be beyond the scope of true parental treatment.^80^ It was the severity of caning which tapped into outrage, with AMMA complaining in the early 1980s that corporal punishment had ‘become associated in the public mind with the relatively new concept … of child abuse’.^81^ The violence of the disciplinary act – all the more pronounced given the longstanding association between beating and sadistic eroticism – served to rupture the *in loco parentis* relationship and the unwritten and implicit social contract on which teachers assumed it to be founded. The response of teacher to these criticisms was to malign the excessive use of corporal punishment by a recalcitrant minority but accept it as a tragic necessity given pupils’ capacity to disrupt the classroom violence or not to heed persistent minor correction. That teachers themselves often declared their abhorrence at being forced to administer the cane was just one recurring feature of this approach, corroborating Gardner’s claim in an analysis of inter-war teachers’ professional subjectivities that while many may not have enjoyed caning their charges, they reacted with hostility to children who refused to accept it.^82^ Indeed, to refuse punishment challenged the parental relationship – in its psychological, legal and symbolic dimensions – upon which teachers’ authority was justified by implying that it had a contractual element and one that could be overridden. It is possible to see in these discourses a changing understanding not only of the teacher’s role *in loco parentis* but equally of the shifting position of the parent, and their expected rights vis-à-vis their children and those adults who have professional contact with them, in post-war Britain.

Abolition campaigners had a long history of defying the authority of the school to cane on the grounds of parental rights (as much as children’s rights). The Committee for the Abolition of Corporal Punishment had felt that there was something problematic in the fact that the ‘Common Law over-rides the position of [a] parent, and places the teacher *in loco parentis*’, although it seems to have stopped short of actively testing this in the courts.^83^ This was not so with STOPP, which took pride in financing court cases, culminating in Campbell and Cosans. The group had been targeting the campaign theme of parental rights since its inception in 1968, with a leaflet from that year describing corporal punishment as ‘making a mockery of the principle of *in loco parentis*’.^84^ However, this also proved a source of tension within the Society. Colin Bagnall, STOPP’s Honorary Secretary and one of the most senior educators in the organisation, even raised his concerns with the limited role of the teaching profession in vetting STOPP propaganda at one of the group’s committee meetings. He felt that STOPP had undertaken ‘sarcastic’ attacks on teachers and individual schools and that, for a nominal ‘Society of Teachers’, it was more interested in parents’ sentiments than the professional well-being of teachers.^85^

The underlying assumption of this was that a society which was originally devised as a pressure group within the teaching profession, and speaking with the authority of members of that profession as much as with the strength of ethical convictions, had become dominated by parental interests. Indeed, the majority of STOPP’s members (and consequently the source of its subscription fees) seem to have been parents rather than educators, without doubt a reflection of the role of CASE – a teacher and parent organisation – in its founding. In early correspondence between STOPP’s founder, Gene Adams, and the campaign, both groups sought to situate the new campaign within a narrative which saw education as a service existing for the benefit of parents.^86^ In one exchange with CASE’s chair, Maurice Plaskow (a parent) invoked the class-based history of compulsory schooling (that, incidentally, expounded in the works of Humphries and Davin) to argue that

the concept of teachers being ‘in loco parentis’ is one which needs rethinking and is a legacy of a charitable and patronising system of education. Teachers are definitely not there in my loco … and if they think they are then they’d better not lay their hands (or anything else) on my children, since I don’t myself.^87^

This is not to suggest that such attitudes enjoyed universal support amongst CASE supporters. Reactions among teaching members were particularly scathing of the plan to encourage children’s guardians to send STOPP-branded pro forma and specimen letters into schools informing them that their right to discipline by force had not been delegated.^88^ Given the legal nature of *in loco parentis*, this action carried no effect whatsoever, other than to ‘stir up’, in the words of one complainant, ‘trouble' and 'distrust’. This correspondent felt that ‘it will be increasingly difficult for many teachers to remain members of CASE’ if the organisation ‘continued to provide financial and material support to STOPP’.^89^ Such campaigning was therefore perceived as a challenge to the argument of teachers’ expertise. Yet, more significantly for this analysis, it exemplified anti-corporal-punishment activists’ efforts to reinterpret the meaning of *in loco parentis* as less a compulsory delegation of parental authority to the teacher and more as a voluntary contractual arrangement between a service user and public servant. The contradiction is highlighted by the fact that while many traditionally-minded Conservative MPs sought (without success) to derail the abolition clause in the 1986 Education Bill, their own government was already planning major structural changes to school administration and organisation with the stated aim of placing a renewed onus on improving ‘choice’ for parents .^90^

## Conclusion

If, as Sascha Auerbach notes about parental opposition to state education in the nineteenth century, the ‘dissonance’ was between those ‘who prioritised the needs and rights of parents from those who prioritised the state’s duty to protect children’, by the late-twentieth century, the issues surrounding school discipline, parents and children seem to have been transfigured into a question of parent versus teacher, rather than parent versus state.^91^ One fairly evident reason for this was growing public support for education as part of the state’s duties to its citizens; another was that teachers become more organised and visible as a comparatively autonomous, middle-class profession, rather than simply as agents of a higher authority. In other words, the issue had become less one of restricting the scope of the state and more one of critiquing and limiting professional power. Hence, STOPP’s campaigning emphasised the brutality of corporal punishment but was equally concerned with challenging the concept of *in loco parentis* itself. Campbell and Cosans, together with the consequent ministerial wrangling over its legal consequences, were merely catalysts in a longer narrative of opposition to corporal punishment and debates around the place of the parent and teacher in the educational system. Gaining ground from a radical fringe in the inter-war period, narratives critical of *in loco parentis*, with reference to corporal punishment, were eventually to attain their aims by merging with a discourse of parental (and consumer) rights towards the end of the century.

Conversely, from the teachers’ perspective, *in loco parentis* went beyond a mere delegation of rights and responsibilities connected with children. It was recognised as part of their professional identity, connected with their self-perception as a group concerned with the welfare of children, and instrumentalised as a strategy for retaining effective disciplinary powers. Teachers understood the classroom as an environment where the maintenance of good order was transactional, based on the perceived need of children to have limits imposed on their behaviour and to have punishment dealt out reasonably to expurgate any sense of lingering guilt.^92^ The benefit of physical punishment, as it was constructed rhetorically, thus became individualised. The central component of the teacher’s claim to a form of professionalised expertise in this settlement hence relied on their being able to understand the child as a holistic entity, to take account of their personalities and problems in a distinctly parental way, while nonetheless balancing these with the well-being of the class as a whole.

Yet, it is revealing that in the range sources examined here (from union and organisation archives to published records), there is no interpolation of these two roles directly. In other words, teachers never examined their *in loco parentis* role through the prism of their own status as parents, despite the fact that a sizeable proportion of male and female teachers by the mid-century were almost certain to have been parents themselves. One explanation for this may well reside in a desire to maintain a division between professional and private life. Yet, this is all the more intriguing when we consider that neither Farley nor Bowley, writing from a more personal perspective, never mention their own family statuses. Instead, they invoke images of abstract fatherhood, just as in the constructions of Steedman’s ‘mothers made conscious’. Unlike women, however, male teachers were never subjected to a marriage bar (which had the simultaneous effect of also banning actual mothers from the profession) and consequently did not have to negotiate what Steedman felt to be the primary contradiction to beset women: the need to illustrate a capacity for maternal affection in the absence of a child of their own.^93^ Another notable omission in the source-base on *in loco parentis* is the fact that children are most often talked about in an abstract and ungendered way – even though the male, working-class teenagers described by Farley constituted the group most likely to receive corporal punishment. Indeed, only in a few rare examples are the pupils receiving punishment identified in explicitly class-based, age-specific and gendered terms. Perhaps most union papers, written in a more impersonal style and seeking to elicit as wide-ranging support for corporal punishment as possible, adopted a deliberately general and homogenising understanding of children. This may be corroborated by the fact that explicit gendering is most common in writings produced by individuals, and most often predominantly male and paternalistic. Finally, the third major absence is the most intriguing of all: the definition of corporal punishment itself. It is only because of STOPP’s campaigning material that we read ‘corporal punishment’ as synonymous with caning as opposed to slapping or smacking. The collapsing of language which takes place, especially given the associations of the latter methods with parental discipline, further reinforced a perceived continuity (at least for teachers) between correction in the home and the school.

Taken together, these silences within the sources may well indicate *in loco parentis*’ rhetorical limitations. Teachers were presumed to enjoy a form of delegated parental authority in which *in loco parentis* embodied a caring role, but this ultimately created a discursive trap for the profession in which parents could claim a right to legitimately refuse the delegation of their powers. This was exploited by abolitionist groups who offered their own cultural narratives of what *in loco parentis* should mean, while teachers (despite their reliance on parental doctrine) seemed reticent to collapse distinctions between professional and private life. The texts analysed here, in debating who should control and influence the classroom moral economy, thus also reveal different and competing conceptions of where the boundary between professional and parent should be drawn in post-1945 Britain.

## Notes

1.
*Report of the Departmental Committee on Corporal Punishment* (London: HMSO, 1938), pp. 35–36. For examples of the contemporary psychological risks: Edward Glover, ‘The psycho-pathology of flogging: a study in the motivation of penal method’; in George Benson and Edward Glover, *Corporal Punishment: an indictment* (London, 1931), pp. 15–29; and Ian Gibson, *The English Vice: beating, sex and shame in Victorian England and after* (London, 1978).

2.
*Report of the Departmental Committee on Corporal Punishment*, p. 34.

3. Peter Mandler, ‘Educating the Nation I: Schools’, *Transactions of the Royal Historical Society*, 24 (2014), pp. 5–28.

4. Moira J Maguire and Séamus Ó Cinnéide, ‘“A good beating never hurt anyone”: the punishment and abuse of children in twentieth-century Ireland’, *Journal of Social History*, 38:3 (2005), pp. 635–652.

5. Stephen Humphries, *Hooligans or Rebels? An oral history of working-class childhood and youth, 1889–1939* (Oxford, 1981); Jonathan Rose, ‘Willingly to school: the working-class response to elementary education in Britain, 1875–1918’, *Journal of British Studies*, 32:2 (1993), pp. 114–138; John Gillis, *Youth and History: tradition and change in European age relations, 1770-present* (London, 1981), p. 174; and Anna Davin, *Growing Up Poor: home, school, and street in London, 1870–1914* (London, 1996), pp. 129–131.

6. See Mary Clare Martin, ‘Church, school and locality: revisiting the historiography of “state” and “religious” educational infrastructures in England and Wales, 1780–1870’, *Pedagogica Historica*, 49:1 (2013), pp. 70–81.

7. Siân Pooley, ‘“All we parents want is that our children’s health and lives should be regarded”: child health and parental concern in England, *c*.1860–1910’, *Social History of Medicine*, 23:3 (2010), pp. 528–548; idem, ‘Parenthood, citizenship and the state in England, *c*.1870–1914’, in Hester Barron and Claudia Siebrecht (eds), *Parenting and the State in Britain and Europe, c.1870–1950: raising the nation* (Basingstoke, 2017), pp. 25–48; Hester Barron, ‘Parents, teachers and children’s well-being in London, 1918–1939’, in idem., pp. 137–160; Sascha Auerbach, ‘“The law has no feeling for poor folks like us!”: everyday responses to legal compulsion in England’s working-class communities, 1871–1904’, *Journal of Social History*, 45:3 (2012), pp. 686–708; idem., ‘“Some punishment should be devised”: parents, children and the state in Victorian London’, *The Historian*, 71:4 (2009), pp. 757–779; and Christine Heward, ‘The class relations of compulsory school attendance: the Birmingham Jewellery Quarter, 1851–86’, *History of Education Quarterly*, 29:2 (1989), pp. 215–235.

8. Barron, ‘Parents, teachers and children’s well-being’, p. 142; and Christine Wall, ‘Picturing an occupational identity: images of teachers in careers and trade union publications 1940–2000’, *History of Education*, 37:2 (2008), pp. 317–340.

9. Deborah Thom, ‘The healthy citizen of Empire or juvenile delinquent?: beating and mental health in the UK’, in Marijke Gijswijt-Hofstra and Hilary Marland (eds), *Cultures of Child Health in Britain and the Netherlands in the Twentieth Century* (Amsterdam, 2003), p. 189; and idem, ‘“Beating children is wrong”: domestic life, psychological thinking and the permissive turn’, in Lucy Delap, Ben Griffin and Abigail Wills (eds), *The Politics of Domestic Authority in Britain since 1800* (Basingstoke, 2009), pp. 261–283.

10. Thom, ‘The healthy citizen of Empire’, p. 207.

11. Thom, ‘Beating children is wrong’.

12. Peter Newell (with anonymous contributors), *A Last Resort? Corporal punishment in schools* (London, 1972), pp. 77–91; Peter Newell, *Corporal Punishment in Schools: Abolition Handbook* (London, undated, [*c*. 1979]), pp. 5–6; and STOPP, *Britain’s Violent Teachers: a dossier of beating reported to STOPP in 1982* (London, 1983); Leila Berg, *Risinghill: death of a comprehensive school* (London, 1968), pp. 9–24; and University of Warwick, Modern Records Centre (MRC), MSS.328/BASW/4/229, British Association of Social Workers (BASW) papers, STOPP booklet, ‘Notes for speakers – revised January 1982’, pp. 4–5.

13. Assistant Masters and Mistresses Association (AMMA), *After Strasbourg: a policy for discipline in schools* (London, n.d. [*c*. 1983]), p. 14.

14. Philip Gardner, ‘The giant at the front: young teachers and corporal punishment in inter-war elementary schools’, *History of Education*, 25:2 (1996), pp. 146–154. For examples of the male teacher as sexual sadist, see Newell, *Abolition Handbook*; and STOPP, *Britain’s Violent Teachers*.

15. Carolyn Steedman, ‘“The mother made conscious”: the historical development of a primary school pedagogy’, *History Workshop*, 20 (1985), pp. 149–163.

16. Laura Tisdall, ‘Inside the “blackboard jungle”: male teachers and male pupils at English secondary modern schools in fact and fiction, 1950 to 1959’, *Cultural and Social History*, 12:4 (2015), pp. 489–507; and Laura Carter, ‘“Experimental” secondary modern education in Britain, 1948–1958’, *Cultural and Social History*, 13:1 (2016), pp. 29–35.

17. Laura Tisdall, ‘Education, parents and concepts of childhood in England, *c*.1945-*c*.1979’, *Contemporary British History*, 31:1 (2017), pp. 24–46; Carter, ‘“Experimental” secondary modern education’; Philip Gardner and Peter Cunningham, *Becoming Teachers: texts and testimonies, 1907–1950* (London, 2004); idem, ‘Oral history and teachers’ professional practice: a wartime turning point?’, *Cambridge Journal of Education*, 27:3 (1997), pp. 331–342; Philip Gardner, ‘The giant at the front: young teachers and corporal punishment in inter-war elementary schools’, *History of Education*, 25:2 (1996), pp. 141–163; Stephen Hussey, ‘The school air-raid shelter: rethinking wartime pedagogies’, *History of Education Quarterly*, 43:4 (2003), pp. 517–539; Martin Lawn, ‘What is the teachers’ job? Work and welfare in elementary teaching, 1940–1945’, in Lawn and Grace (eds), *Teachers*, pp. 50–64; Carolyn Steedman, ‘Prisonhouses’, in Lawn and Grace (eds), *Teachers*, pp. 117–129.

18. Asher Tropp, *The School Teachers: the growth of the teaching profession in England and Wales from 1800 to the present day* (London, 1957); Martin Lawn, *Servants of the State: the contested control of teaching, 1900–1930* (Lewes, 1987), pp. 117–138; Sarah King, ‘Feminists in teaching: the National Union of Women Teachers, 1920–1945’, in Martin Lawn and Gerald Grace (eds), *Teachers: the Culture and Politics of Work* (Lewes, 1987), pp. 31–49.

19. Laura King, *Family Men: fatherhood and masculinity in Britain, c.1914–1960* (Oxford, 2015), pp. 73–75.

20. Ibid., pp. 60–61, 75.

21. Ibid, p. 74.

22. The phrase achieved prominence with E P Thompson’s much-cited essay ‘The Moral Economy of the English Crowd’ in 1971, where it was employed to contextualise the demands by the rural and urban poor in the eighteenth century for the ‘just price’ of bread: E P Thompson, ‘The moral economy of the English crowd in the eighteenth century’, *Past and Present*, 90 (1970), pp. 76–136. For a brief history of the term’s usage prior to this, see Norbert Götz, ‘“Moral economy”: its conceptual history and analytical prospects’, *Journal of Global Ethics*, 11:2 (2015), pp. 147–162.

23. See, by way of example, two of the pieces from a recent (2015) journal issue devoted to ‘moral economy’: Götz, ‘Moral economy’; and Johanna Siméant, ‘Three bodies of moral economy: the diffusion of a concept’, *Journal of Global Ethics*, 11:2 (2015), pp. 163–175.

24. Lorraine Daston, ‘The moral economy of science’, *Osiris*, 10 (1995), p. 4.

25. For some discussion of these topics: Laura Tisdall, ‘“That was what life in Bridgeburn had made of her”: reading the autobiographies of children in institutional care in England, 1918–46’, *Twentieth Century British History*, 24:3 (2013), pp. 351–375; Carol Dyhouse, *Girl Trouble: panic and progress in the history of young women* (London, 2014), pp. 116–117; and Rob Boddice, ‘*In loco parentis*? Public-school authority, cricket and manly character, 1855–62’, *Gender and Education*, 21:2 (2009), pp. 159–172.

26. Barrell, *Legal Cases for Teachers* (London, 1970), p. 148.

27. Ibid., pp. 148, 150.

28. Ibid., p. 216.

29. The two foundation cases in modern case law are arguably *R v Hopley* (1860) 2 F & F 202 and *Mansell v Griffin* (1908) 1 KB 160, 947. See Barrell, *Legal Cases for Teachers*, pp. 216–222.

30. Carol Dyhouse, *Students: a gendered history* (Abingdon, 2006).

31. Humphries, *Hooligans or Rebels?*; and Rose, ‘Willingly to school’.

32. Boddice, ‘*In loco parentis*?’.

33. Barron, ‘Parents, teachers and children’s well-being’.

34. Ethel Mannin, *Common-Sense and the Child: a plea for freedom* (London, 1932); idem, *Common-Sense and the Adolescent* (London, 1937); A S Neill, *The Problem Child* (London, 1929); idem, *The Problem Parent* (London, 1932); idem, *The Problem Teacher* (London, 1939); and Arthur B Allen, *The Psychology of Punishment: the new school discipline* (London, 1936).

35. The only evidence for its existence that I have found are in correspondence, pamphlets and press cuttings of the left-wing publisher, Victor Gollancz. Gollancz signed a letter published in the *Times* newspaper by the group in 1946 and also contributed some funds to campaigns during the late-1940s. See the file of correspondence: MRC, MSS.157/3/CO/1/1-28, Victor Gollancz papers.

36. Department for Education and Science, *Children and their Primary Schools: a report of the Central Advisory Council for Education (England)*, Vol. 1: *The Report* (London: HMSO, 1967), pp. 271–272.

37. STOPP asserted that many authorities had not introduced sufficient limitations or regulations, let alone prohibitions, by the time of the Campbell and Cosans ruling in 1982: MRC, MSS.144/5/9, Frank Crump papers, ‘STOPP News’, February–March 1984.

38.
*Campbell and Cosans v United Kingdom*, 25 February 1982, European Court of Human Rights. For the responses of teachers’ unions, see MRC, MSS.292E/815.94/1 Box E151, Trades Union Congress (TUC) papers, Letter from Nigel de Gruchy (NAS-UWT) to Roy Jackson (NUT), 10 December 1984; MRC, MSS.179/2/29/8, National Union of Teachers (NUT) papers, Intercommunication from Hugh Pierce to Janet Rowe, 22 September 1983; MRC, MSS.179/2/29/8, NUT papers, ‘Report’, 2 September 1983; and AMMA, *After Strasbourg*. For a legal analysis of the Campbell and Cosans ruling and its impact on case law, please refer to Dympna Glendenning, *Education and the Law* (Dublin, 2012), pp. 433–435.

39. The National Archives of the United Kingdom (TNA), ED 269/198, ‘Corporal punishment: European Court of Human Rights Judgement. Implications for UK Govt’, 1983. See also TNA, ED 269/377-378.

40. MRC, MSS.292E/815.94/1 Box E151, TUC papers, letter, Nigel de Gruchy (NAS-UWT) to Roy Jackson (NUT), 10 December 1984.

41. MRC, MSS.639/11/53/3, NUT papers, Circular 170/82, 22 April 1982. This was in response to the government’s policy consultation in the aftermath of the Campbell and Cosans ruling; the proposals would have allowed individual parents to opt their children out of corporal punishment, which the union believed would risk creating inequalities among the members of a given class: MRC, MSS.639/11/53/7, NUT papers, ‘NUT response to DES consultative document “Corporal punishment in schools”, 1983’.

42.
*Education (No. 2) Act*, 1986, section 47.

43. Mandler, ‘Schools’.

44. Mathew Thomson, *Psychological Subjects: identity, culture and health in twentieth-century Britain* (Oxford, 2006), pp. 109–139; Michal Shapira, *The War Inside: psychoanalysis, total war and the making of the democratic self in post-war Britain* (Cambridge, 2013); and King, *Family Men*, pp. 91–116.

45. John Bowlby, *Maternal Care and Mental Health: a report prepared on behalf of the World Health Organisation as a contribution to the United Nations programme for the welfare of homeless children* (Geneva, 1952); Denise Riley, *War in the Nursery: theories of the child and mother* (London, 1983); Shapira, *The War Inside*; and King, *Family Men*, pp. 57–68.

46. Allen, *The Psychology of Punishment*, p. 34.

47. Tisdall, ‘Inside the “blackboard jungle”’.

48. Richard Farley, *Secondary Modern Discipline: with special reference to the ‘difficult’ adolescent in socially depressed industrial areas* (London, 1960), pp. 97–98. On the theme of counselling in the classroom, see Alick Holden, *Teachers as Counsellors* (London, 1969).

49. Rex Bowley, *Teaching Without Tear: a guide to teaching technique* (London, 1973 [1961]), p. 26. This book went through at least four editions after 1961, but the advice on corporal punishment remained unchanged.

50. King, *Family Men*, pp. 69–70.

51. Farley, *Secondary Modern Discipline*, pp. 68–70. See also King, *Family Men*, pp. 65–68.

52. Tisdall, ‘Inside the blackboard jungle’, pp. 495–498; and Tisdall, ‘Education, parenting and conceptions of childhood’. For examples in psychological texts, see J A Hadfield, *Childhood and Adolescence* (London, 1962), pp. 109–114; and Agatha Bowley, *Modern Child Psychology* (London, 1948), p. 8.

53.
*Report of the Departmental Committee on Corporal Punishment*, p. 35.

54. Home Office, *Report of the Departmental Advisory Council on the Treatment of Offenders* (London: HMSO, 1960). Otherwise known as the ‘Barry Report’, this was established by the government to examine the case for a reintroduction of judicial birching. Like Cadogan, it ultimately recommended against the practice.

55. MRC, MSS.179/EDU/3/12/52, NUT papers, ‘Corporal punishment: memorandum of evidence submitted by the Executive of the Union to the Home Office Advisory Council on the Treatment of Offenders’, 2 April 1962.

56. MRC, MSS.179/EDU/3/12/52, NUT papers, Intercommunication, K Wormald to Education Department, 30 April 1962.

57. National Union of Teachers, *Plowden: the Union’s comments on some of the major issues of the Plowden Report* (Manchester, n.d. [1967]), p. 17.

58. MRC, MSS.59/3/1/13, Association of Assistant Mistresses papers, letter, Secretary of the AAM to M G Woolley, 10 February 1976.

59. London, Institute of Education Archives (IoE), PL/6/4, Plowden papers, Plowden Working Party 4, Paper 104, ‘The Child and the Community: values, discipline and behaviour’, Appendix E, National Association for Mental Health, ‘Memorandum on the effect of corporal punishment on primary school children’, 1965, p. 2.

60. IoE, PL/6/4, Plowden papers, Plowden Working Party 4, Paper 104, ‘The Child and the Community: values, discipline and behaviour’, Appendix D, ‘Evidence from the British Psychological Society’, n.d., p. 1.

61. Farley, *Secondary Modern Discipline*, p. 65–66.

62. Charles W Valentine, *The Difficult Child and Problem of Discipline* (London, 1940), p. 82.

63. Newell, ‘Preface’, in *A Last Resort?*, p. 7.

64. Newell, *Abolition Handbook*, p. 24.

65. National Foundation for Educational Research, *A Survey of Rewards and Punishments in Schools: NFER Publication No. 3* (London, 1952).

66. IoE, PL/6/4, Plowden papers, Plowden Working Party 4, Paper 104, ‘The Child and the Community: values, discipline and behaviour’, Appendix B, NUT submission, pp. 1–2.

67. Ibid., p. 2.

68. MRC, MSS.179/EDU/3/12/52, NUT papers, untitled draft of document on corporal punishment, undated, p. 1.

69. MRC, MSS.328/BASW/4/229, BASW papers, NUT leaflet, ‘Corporal punishment: the case for alternatives’, n.d. [*c*. 1983], p. 7.

70. IoE, PL/6/4, Plowden papers, Plowden Working Party 4, Paper 104, ‘The Child and the Community: values, discipline and behaviour’, Appendix C, NAS submission, January 1965.

71. Joanna Bourke, *Dismembering the Male: Men’s bodies, Britain and the Great War* (London, 1996), pp. 192–198.

72. MRC, MSS.292E/815.94/1 Box E151, TUC papers, letter, Terry Casey to Shirley Williams, 15 July 1977.

73. AMMA, *After Strasbourg*, p. 3.

74. Ibid., p. 4.

75. Ibid., p. 24.

76. MRC, MSS.179/EDU/3/12/52, NUT papers, typescript, ‘Deputation on corporal punishment’, 21 March 1968.

77. Gardner, ‘The giant at the front’, p. 141.

78. Farley, *Secondary Modern Discipline*, p. 28 (word ‘censored’ in original).

79. Ibid., pp. 54–55.

80. Anonymous contributor, in *A Last Resort?*, p. 129.

81. AMMA, *After Strasbourg*, p. 9. For a more detailed discussion on concerns about paedophilia in the 1970s, see Mathew Thomson, *Lost Freedom: the landscape of the child in the post-war British settlement* (Oxford, 2013), pp. 153–183.

82. Gardner, ‘The giant at the front’, pp. 157–159.

83. MRC, MSS.157/3/CO/1/8, Victor Gollancz papers, pamphlet, ‘The Committee for the Abolition of Corporal Punishment in Schools’, n.d. [*c*. 1946], unpaginated.

84. MRC, MSS.378/BASW/2/147, BASW papers, STOPP pamphlet, enclosed with letter from Colin Bagnall to BASW, 11 September 1977.

85. IoE, STO/1/2/9, STOPP papers, Executive committee minutes, note from Colin Bagnall, ‘STOPP: Control by Teachers’, 1 October 1981.

86. MRC, MSS.236/1982/31, Campaign for the Advancement of State Education (CASE) papers, letter, Barbara Bullivant to Gene Adams, 18 March 1972.

87. MRC, MSS.236/1982/31, CASE papers, letter, Gene Adams to Maurice Plaskow, 23 April 1968; MSS.236/1982/31, CASE papers, letter, Plaskow to Adams, 25 April 1968.

88. MRC, MSS.236/1982/31, CASE papers, copy of STOPP sample letter, 22 August 1972.

89. MRC, MSS.236/1982/31, CASE papers, letter, M Roberts to Barbara Bullivant, 3 December 1972.

90. TNA, ED 269/198, ‘Corporal punishment: European Court of Human Rights Judgement. Implications for UK Govt’, 1983.

91. Auerbach, ‘Some punishment should be devised’, p. 172.

92. See Deborah Thom’s assertion about a resurgence of narrow behaviourist understandings of reward and punishment at the end of the twentieth century: Thom, ‘Beating children is wrong’, pp. 279–281. A ‘relations’ and ‘systems’ approach to classroom discipline also influenced a variety of pedagogical textbooks in the early 1980s: Keith Topping, *Educational Systems for Disruptive Adolescents* (London, 1981); Bill Gillham (ed.), *Problem Behaviour in the Secondary School: a systems approach* (London, 1981); and David Galloway, Tina Ball, Diana Blomfield and Rosalind Seyd, *Schools and Disruptive Pupils* (Harlow, 1982).

93. Steedman, ‘The mother made conscious’.

